# The Avoidance of Purine Stretches by Cancer Mutations

**DOI:** 10.3390/ijms252011050

**Published:** 2024-10-15

**Authors:** Aleksandr V. Vikhorev, Ivan V. Savelev, Oksana O. Polesskaya, Michael M. Rempel, Richard A. Miller, Alexandre A. Vetcher, Max Myakishev-Rempel

**Affiliations:** 1 DNA Resonance Research Foundation, San Diego, CA 92111, USA; 2 OAK, Inc., Grants Pass, OR 97526, USA; 3 Institute of Pharmacy and Biotechnology (IPhB) of Peoples’ Friendship University of Russia n.a. P. Lumumba (RUDN), 6 Miklukho-Maklaya St, 117198 Moscow, Russia; 4 Institute for Bionic Technologies and Engineering, I.M. Sechenov First Moscow State Medical University, 2-4 Bolshaya Pirogovskaya Str., 119991 Moscow, Russia

**Keywords:** purine stretches, cancer development, aromaticity, delocalization, double helix, enol, amine, tautomers, quantum-chemical models, flanking sequence, mutation susceptibility

## Abstract

Purine stretches, sequences of adenine (A) and guanine (G) in DNA, play critical roles in binding regulatory protein factors and influence gene expression by affecting DNA folding. This study investigates the relationship between purine stretches and cancer development, considering the aromaticity of purines, quantified by methods like Hückel’s rule and NICS calculations, and the importance of the flanking sequence context. A pronounced avoidance of long purine stretches by typical cancer mutations was observed in public data on the intergenic regions of cancer patients, suggesting a role of intergenic sequences in chromatin reorganization and gene regulation. A statistically significant shortening of purine stretches in cancerous tumors (*p* value < 0.0001) was found. The insights into the aromatic nature of purines and their stacking energies explain the role of purine stretches in DNA structure, contributing to their role in cancer progression. This research lays the groundwork for understanding the nature of purine stretches, emphasizing their importance in gene regulation and chromatin restructuring, and offers potential avenues for novel cancer therapies and insights into cancer etiology.

## 1. Introduction

Purine stretches, contiguous sequences of adenine and guanine nucleotides in DNA, have long been recognized to be involved in genomic regulation. These stretches can serve as binding sites for regulatory protein factors involved in transcription, replication, and recombination [1,2,3,4,5,6]. Moreover, they may influence gene expression by affecting DNA folding and determining the accessibility of specific regions to cellular regulatory machinery.

Our research focuses on purine stretches due to their unique electrodynamic properties. Our models of electron delocalization indicate that purine stretches exhibit significantly stronger electron delocalization compared to other DNA sequences. This enhanced delocalization suggests that purine stretches may possess biological functions through yet undiscovered mechanisms, potentially influencing chromatin organization and gene regulation on a broader scale than previously understood.

The importance of purine stretches was further emphasized by the concept of flanking sequence context in cancer mutations [7]. These flanking sequences surrounding a specific mutation influence both the occurrence and type of mutations. The flanking sequence can affect DNA stability and conformation, with changes in charge delocalization along these sequences potentially altering DNA function. Remarkably, these effects can extend up to 2000 nucleotides in a “distance-decaying” relationship [7], highlighting the far-reaching impact of local sequence composition on genomic function.

To investigate the functional importance of purine stretches in cancer, we utilized the COSMIC cancer database. This comprehensive resource serves as an excellent substitute for experimental studies, offering paired tumor and healthy tissue complete genome comparisons from thousands of patients. The COSMIC database is particularly valuable because each tumor represented within it is the result of clonal cancer selection, effectively capturing the functional selection of cancer-related mutations.

The mutations shared across multiple patients in the COSMIC database are typically those that have evolved during clonal expansion, enabling tumors to evade immune control and redirect the body’s resources. By analyzing those mutations that are affecting purine stretches, we can gain insights into the functional importance of purine stretches. This method allows us to bridge the gap between theoretical models of electron delocalization and real-world genomic alterations observed in cancer patients, potentially uncovering new mechanisms of chromatin folding and gene regulation.

To understand the biochemical and biophysical function of purine stretches, it helps to consider the fusion and delocalization of pi electrons in purine stretches, as well as the aromaticity of purines. Purines (nucleotides A and G) have a fused double ring consisting of a hexagon fused with a pentagon. Aromaticity occurs via the fusion of pi electrons into a ring, such as in benzene, and results in their delocalization within a ring. Purines A and G having two aromatic rings (hexagon and pentagon) are more aromatic than pyrimidines C and T, which contain only a single hexagon. The order of aromaticity of the four nucleotides in DNA (adenine, guanine, cytosine, and thymine) is A > G > C > T [8,9,10].

Quantifying the extent of aromaticity in nucleotides is a challenging task, as there is no universally accepted definition or method for measuring aromaticity. However, here are a few potential surrogate measures that have been proposed and used in the literature to assess the aromatic character of nucleotides:

1. Hückel’s rule: This rule, developed by Erich Hückel, states that an aromatic molecule must have a planar ring with 4n + 2π electrons, where n is a non-negative integer. Based on this rule, A and G are considered more aromatic than C and T because they have more π electrons in their ring structures [11].
2. Nucleus-independent chemical shift (NICS) calculations: The NICS values of nucleotides can be calculated using quantum chemical methods and can be used as a surrogate measure of their aromaticity. A and G are calculated to have lower NICS values compared to C and T, indicating their more aromatic nature [9].

In addition to base pairing, it is well known that DNA bases stick to each other in the base stack in the double helix in water. This stacking of DNA bases is based on their aromaticity and is responsible for the helical structure of DNA in water. Specifically, the sugar–phosphate backbone is negatively charged and, accordingly, aims to stretch in a straight line due to the repulsion of negative charges of phosphates. These phosphates are hydrophilic and well-hydrated. The inner part of the base stack is hydrophobic, leading to the minimization of its bordering surface with water. Without the sugar–phosphate backbone, it would form a globule. However, due to the covalent bonding of the base stack to the backbone, a balance is struck. While the bases aim to minimize their contact surface with water, the backbone aims to extend as long as possible, ultimately resulting in a stable and geometrically perfect double-helical structure.

The effect of stacking as the longitudinal attraction of the nucleotide occurs due to the interaction of pi electron rings of aromatic carbon-nitrogen rings in the bases [12]. Stacking energy between bases is approximately and respectively noted as follows: purine–purine = 2–3 kcal/mol, purine–pyrimidine = 1–2 kcal/mol, and pyrimidine–pyrimidine = 0.5–1 kcal/mol [13]. DNA melting and other spectroscopic studies suggest that the aromatic electrons of purines are delocalized by extended conjugation over the stacked bases [14]. Charge transfer experiments also suggest aromatic electron delocalization along stacked purines in purine stretches [15,16].

There is no consensus in the literature on the electron behavior in purine stretches. To shed light on the matter, it is crucial to consider that electrons are best explained by quantum chemical models. The Schrodinger wave function, for instance, determines the probability of an electron’s location in space—even a highly localized electron has a small chance of being found elsewhere. Hence, the question of electron delocalization should be approached quantitatively. It is well-established that the electrons in the aromatic rings of purines are delocalized within each pi ring. Due to stacking, these pi rings fuse, resulting in merged electron clouds. The electrons of adjacent stacked purine bases are likely delocalized across at least these two bases. However, the extent of this delocalization and whether electrons can freely move or tunnel along the purine stretch spanning multiple bases is still unclear [16,17,18,19,20]. DNA sequence elements, such as poly-A tracts and CpG islands, also play critical roles in chromatin organization and gene regulation. Poly-A tracts are known to exclude nucleosomes, creating regions of open chromatin [21,22]. CpG islands, often found in gene promoters, can be methylated to influence gene expression [23,24]. Understanding these sequence-dependent structural features is crucial for studying how purine stretches affect chromatin dynamics and gene regulation. This study builds on existing knowledge of sequence-dependent DNA shape and its implications for chromatin organization.

The role of electron charge transfer in DNA repair and protection is also of practical importance, as the delocalization of electrons in purine stretches protects against chemical and radiation damage [25]. Indole structures possess special aromaticity due to their unique molecular geometry and bonding pattern. The atoms in the ring are arranged in such a way that they follow Hückel’s rule for aromaticity, which states that a planar, fully conjugated ring system with 4n + 2 pi electrons (where n is a non-negative integer) exhibits aromatic properties. The nitrogen atom in the indole ring also contributes to its stability by creating a conjugated system with the adjacent aromatic ring. This results in a molecule with high stability, making it an important component in many biologically active compounds. Purines (A, G) can exist in the enol-amine form (often referred to as the imidazole form) and keto-imine forms. The enol-amine form is more stable and biologically significant than the keto-imine form. This enhanced stability is attributed to the fully conjugated ring system in the enol-amine form, which adheres to Hückel’s rule and becomes aromatic. The presence of a delocalized pi electron cloud within this fully conjugated ring system results in an aromatic molecule. In contrast, the keto-imine form lacks full conjugation in its ring system due to a broken double bond between the nitrogen and carbon atoms, rendering it non-aromatic. The pi electron cloud in this form is more localized.

**Figure 1:**
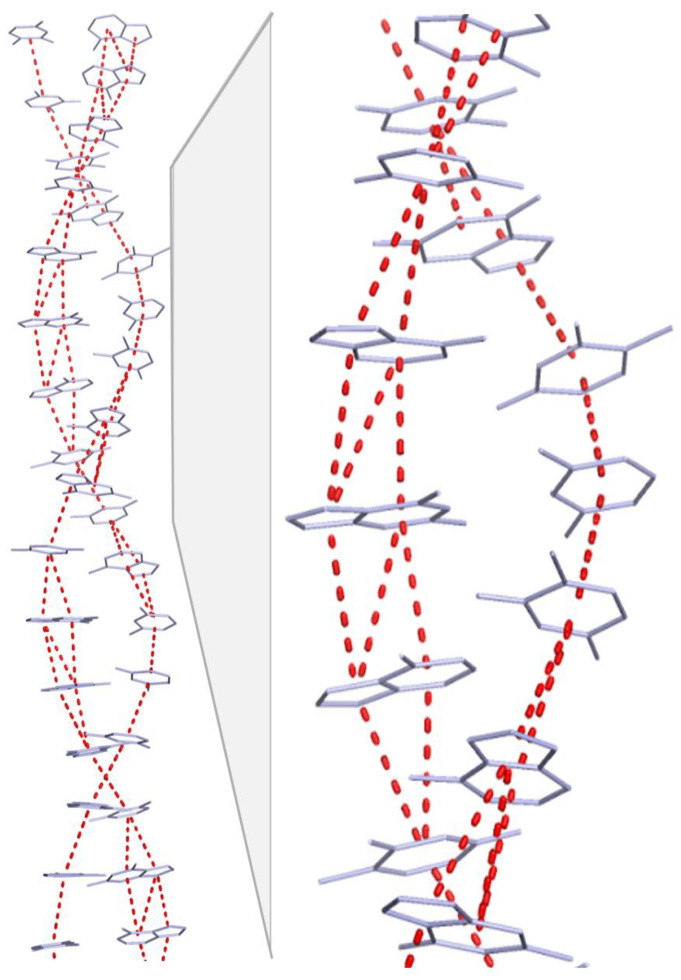
Base stack with electron wires. In the double helix base stack, π–π interactions were visualized automatically in PyMOL (Schrödinger, LLC, New York, NY, USA) to illustrate base stacking, dashed red lines. Note the higher extent of base stacking between consecutive purines (which have two fused rings) compared to pyrimidines (which have one ring).

The DNA structure likely enables long-distance electron transfer via stacked pi electron rings, primarily in purines, acting as “electron wires”. This is due to pi–pi interactions among the overlapping, delocalized electrons in these aromatic rings. Complementarity in DNA ensures that a stretch of purines (each with two pi rings) in one strand always pairs with a stretch of pyrimidines (single pi ring) in the complementary strand. This arrangement inherently forms a “double wire” in the purine strand and a “single wire” in the corresponding pyrimidine stretch, facilitating electron transport (Figure 1).

Potential roles for stretches of purines in DNA are intriguing to consider, albeit they currently remain underexplored. The inherent “double wire” electron transport system in these purine stretches, due to their stacked pi electron rings, might theoretically enable long-range electron transfer, which could be implicated in processes such as DNA repair. Similarly, the positioning and presence of these purine sequences could hypothetically influence the three-dimensional structure of DNA, potentially affecting gene regulation. They might also play a part in moderating the interactions between DNA and various proteins or even be implicated in cellular redox reactions. These are conjectural roles, and the precise functions of purine stretches in terms of electron transport within DNA remain open for exploration. The concept that purine stretches or “wires” in intergenic regions could encode functional information presents a fascinating hypothesis in the context of gene regulation and chromatin dynamics, thereby potentially influencing cellular responses to environmental cues.

Recent biophysical studies and molecular dynamics simulations have provided insights into the potential mechanisms by which purine stretches may influence gene regulation and contribute to cancer development. Multiscale simulations of DNA have revealed that sequence-dependent structural properties can significantly affect DNA–protein interactions and chromosomal organization [26]. Specifically, purine-rich sequences have been shown to alter DNA flexibility and curvature, potentially modifying the binding affinity of transcription factors and other regulatory proteins [27]. Furthermore, studies on core promoter regions have demonstrated that the thermally induced dynamics of DNA, which are influenced by base composition, can play a crucial role in transcription initiation [28]. In the context of chromatin structure, the presence of purine stretches may affect nucleosome positioning and stability, thereby influencing gene accessibility [29]. Additionally, the unique structural properties of purine-rich sequences could impact DNA supercoiling, which has been shown to have significant effects on chromatin architecture and gene expression [30]. Our findings of purine stretch alterations in cancer align with these mechanistic insights, suggesting that disruptions in purine stretch integrity could perturb normal regulatory processes through changes in DNA structure, flexibility, and protein–DNA interactions.

**Figure 2:**
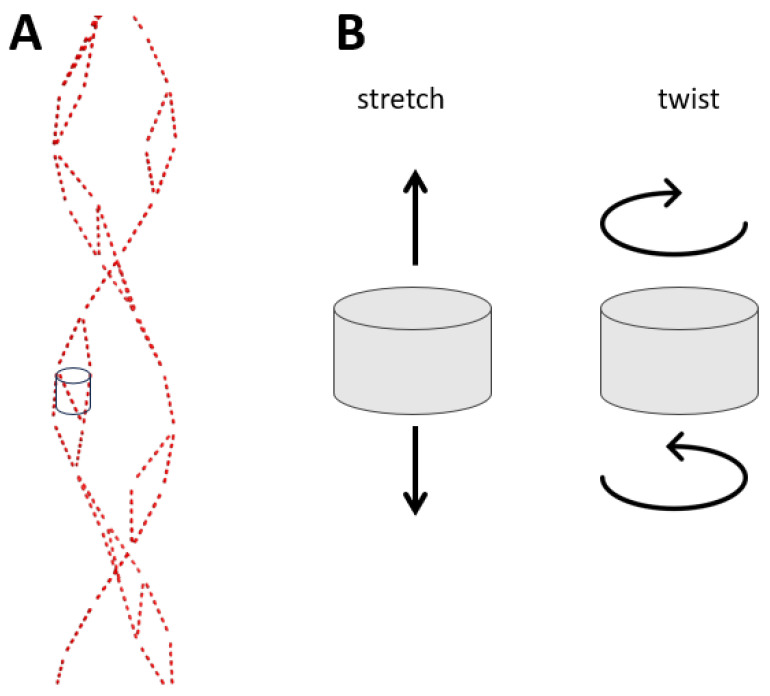
Possible longitudinal (stretch) and twisting oscillations in aromatic electron wires in purine stretches. (**A**) Electron wires from Figure 1, dashed red lines. (**B**) Possible longitudinal and twisting oscillations.

Therefore, we hypothesized that purine stretches play a special functional role in intergenic regions. To test this hypothesis, the most accessible approach involves examining the role of purine stretches in genomic sequences in functional genomic data. We undertook computational analysis to consider purine stretches as electron cloud containers, functioning as oscillators or resonators. The dimensions of these oscillators or resonators would dictate their frequency of oscillation, which could involve stretching or twisting as shown in Figure 2. The function and frequency of these oscillations would vary based on the length of the purine stretch. In this study, we focused on assessing the significance of purine stretch length using publicly available functional genomic data.

**Figure 3:**
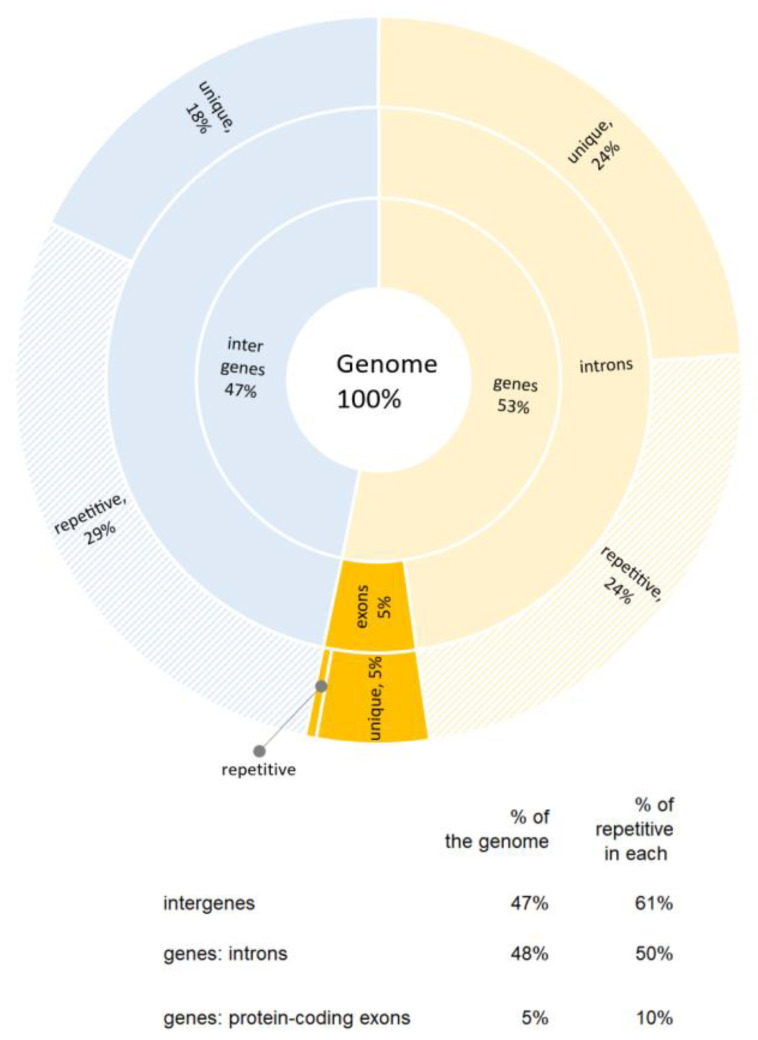
Proportion of exons, introns, and intergenic regions in the human genome summarized from Ensembl Annotation (release 108, October 2022).

To produce a better computational signal and reduce confounding influences, we focused our analysis on the areas of the genome where we expected the function of purine stretches to be most revealed and unhindered by other functions. Since protein-coding exons are under strong pressure to produce correct proteins, we do not expect these to reveal the function of purine stretches in dynamic chromatin self-organization. Therefore, we explored this in noncoding parts of the genome (to be more exact in non-protein-coding parts of the genome). To illustrate the proportions of major types of genome annotations, we calculated the coverage of the genome by coding and noncoding parts based on the current genome assembly (Figure 3).

As shown in Figure 3, we found that protein-coding sequences (protein-coding exons) occupied 5% of the genome. The remaining 95% are not coding for proteins. In addition, 10% of protein-coding exons are repetitive (as defined by Repeat Masker). Genes occupied 53% of the genome. In addition to the above-mentioned protein-coding exons, which make up 5% of the genome, genes also contain introns that comprise 48% of the genome. Half of the intronic sequence is repetitive (24% of the genome). Intergenic sequences (called here intergenes) comprised 47% of the genome, over half (61%) of which are repetitive (29% of the genome). Additionally, it is estimated that less than 50% of genes are expressed in the same somatic cell at the same time.

Currently known functions of introns include enhancing gene expression by increasing the efficiency of transcription and enabling alternative splicing, leading to the production of multiple protein isoforms from a single gene. Introns can also serve as regulators of gene expression at various levels and influence the positioning of nucleosomes, thereby affecting chromatin structure and accessibility in a sequence-specific manner. They play a role in maintaining genome integrity through their involvement in recombination and repair processes, and some even exhibit ribozyme activity. Introns may host sequences that give rise to microRNAs (miRNAs), contributing to post-transcriptional regulation, and facilitating evolutionary innovation by allowing greater flexibility in exon shuffling. Moreover, they can serve as a source for the creation of new genes through evolutionary mechanisms and may influence translation efficiency through their impact on the splicing and translation process. We also suggest that the main functions of introns are still undiscovered. We find it very unlikely that the known functions of introns justify their length. Since the cells spend much of their resources on maintaining introns that occupy 48% of the genome, introns must be playing yet unknown important functions. Since introns are transcribed and this RNA is likely playing important known and yet unknown functions, the sequences of introns are under evolutionary pressure in a different way than intergenic sequences. In our various analyses, we noticed very pronounced differences in DNA patterns between introns and intergenes. Our initial analyses demonstrated that signals from purine stretches were much stronger in intergenic sequences than in introns, so the main part of this study was focused on intergenes. In this study, we will present evidence that purine stretches are statistically shorter in cancerous tissues compared to normal tissues. We will also discuss the significant role of purine stretches in the continuous reorganization of chromatin and gene regulation. These findings highlight the crucial role of purine stacking in maintaining DNA structure and function and suggest that disruptions in these interactions may contribute to cancer development.

While cancer formation involves various factors, such as increased DNA damage, replication errors, and impaired DNA repair, our study focuses on the end result of these processes. The majority of tumors analyzed in our study represent the outcome of cancer evolution and clonal selection, encompassing both the initiation phase based on initial mutagenesis and the progression to metastasis. Our analysis specifically examines the effect of this clonal evolution on purine stretches in intergenic regions. Since purine stretches are located in intergenic regions, and since they are under cancer evolution pressure, they must be functionally important. They most likely play a significant role in regulation of gene expression and chromatin folding.

## 2. Results

### 2.1. Study Design

In the background section, we explained that purine stretches harbor a collectively shared aromatic electron ring stack that possesses unique electromagnetic properties. We hypothesized that purine stretches in intergenic regions play a significant function in the process of continuous sequence-specific restructuring of chromatin and perform complex gene regulation functions. Here, we ventured to test this hypothesis using publicly available functional genomic data.

**Figure 4:**
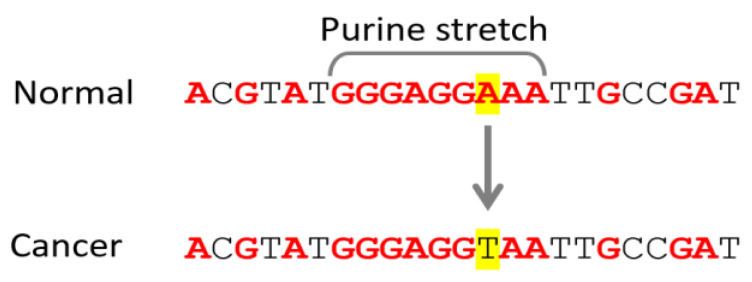
An example of shortening of purine stretch length in cancer. A purine stretch is defined as a string of at least 3 purines (A, G) framed on both sides by at least one pyrimidine (C, T). A mutation that substitutes a purine with a pyrimidine shortens a purine stretch. We looked for the purine stretches in both DNA strands.

The Cosmic dataset (https://cancer.sanger.ac.uk/cosmic (accessed on 6 October 2024)) is an ideal dataset for such analysis. It is curated and contains high-quality data. We selected 4.7 M mutations in promoter regions. Each mutation is represented by a pair of alleles for each patient—an allele from the normal healthy tissue (Normal, Ref_Chain, reference chain) and an allele from a cancer tumor (Cancer, Alt_Chain, alternative chain). The fact that cancer and normal alleles are present in the same patient and the reference allele serves as a healthy control within the same patient makes these data highly informative and reduces the signal-to-noise ratio. Here, normal DNA from each patient serves as a control to the tumor from the same patient. Also very helpful is that the cancer tumors were rigorously characterized. We used the sequence surrounding the mutations to measure the length of the purine stretches (Figure 4).

Since many of the cancer mutations are typical and essential for the clonal evolution of cancer, if they have a preference for shortening or lengthening purine stretches, this would provide evidence for the functional importance of purine stretch length. Therefore, we analyzed the influence of cancer mutations on the length of purine stretches. In the initial analyses, we separated all genomic sequences into exonic (for protein-coding exons), intronic, and intergenic sequences. Our initial analyses demonstrated that all influences of cancer mutations on purine stretch lengths were substantially more pronounced in intergenic regions than in genes (introns and exons). We interpreted this in such a way that genes that are transcribed into RNA are under evolutionary pressure to perform functions related to RNA, while intergenic regions (intergenes) likely function to guide continuous reorganization of chromatin in a sequence-specific manner. Therefore, we limited our consequent analyses to intergenic regions.

### 2.2. On Average, Cancer Mutations Tend to Avoid Purine Stretches

**Figure 5:**
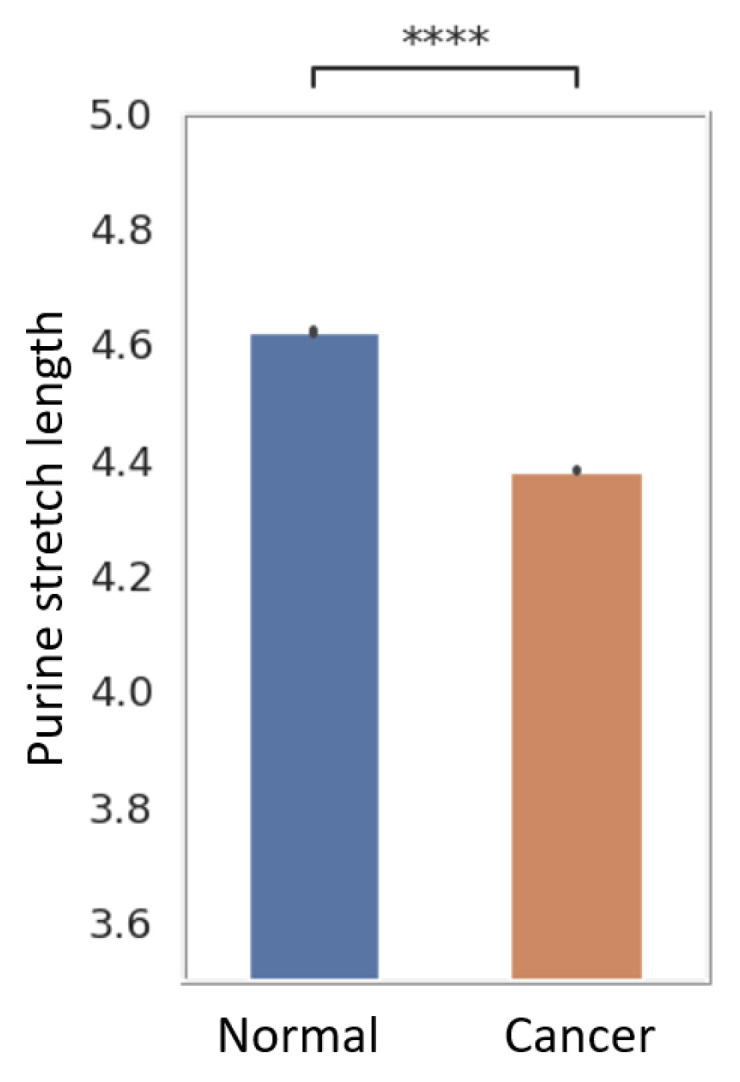
Intergenic purine stretches are shortened in cancer. Mutations in the dataset were represented by a pair of alleles for each patient—an allele from the normal healthy tissue and one from the cancer tumor. Purine_stretch_length was averaged for all mutations and plotted on the Y axis. Purine_stretch_length was defined as the length of the purine stretch containing the mutation in either strand of DNA and framed (interrupted) by pyrimidines. The average Purine_stretch_length was observed to be shorter in cancer than in normal tissue (**** signifies *p* value < 0.0001).

The dataset contained a pair of alleles for each patient—an allele from the normal healthy tissue and an allele from a cancer tumor. The Purine_stretch_length was averaged for all SNPs and plotted on the Y axis. Purine_stretch_length was defined as the length of the purine stretch encoding the mutation in either strand of DNA and limited by pyrimidines. A total of 4.7 M mutations were analyzed. Of these, the most mutations are of the break type (1.5 M), followed by the join type (1.1 M), and mutations that do not change the Purine_stretch_length (2.1 M). For each allele (normal or tumor), the length of the purine stretch, which includes the mutated nucleotide, was measured irrespective or the type of the break/join/no change type and the resulting lengths were combined and averaged. The average Purine_stretch_length was observed to be shorter in cancer than in normal tissue (*p* value < 0.0001 by paired *t* test), as seen in Figure 5. A nonparametric Mann–Whitney test also showed high significance (*p* < 0.001).

### 2.3. Mutations of the Purine Stretches in Promoters

Among intergenic regions, gene promoters upstream of the transcription start site are known to be functional and conserved in evolution since they contain a substantial part of the information of when and where the gene is expressed.

Promoter regions play a crucial role in gene regulation, harboring various regulatory elements that control gene expression [31]. Our focus on purine stretches in promoter regions (400 bp upstream of transcription start sites) builds upon this understanding. Other important promoter elements include CpG islands [32], TATA boxes [33], and initiator (Inr) elements [34]. Transcription factor binding sites (TFBS) are also critical for gene-specific regulation [35].

Purine stretches represent a distinct class of sequence features, characterized by their A and G composition rather than specific motifs. Our findings on purine stretch alterations in cancer complement studies on other promoter elements, such as changes in CpG island methylation [36]. The avoidance of purine stretches by cancer mutations suggests a novel functional importance, potentially paralleling the role of other conserved promoter elements in maintaining proper gene regulation [37].

**Figure 6:**
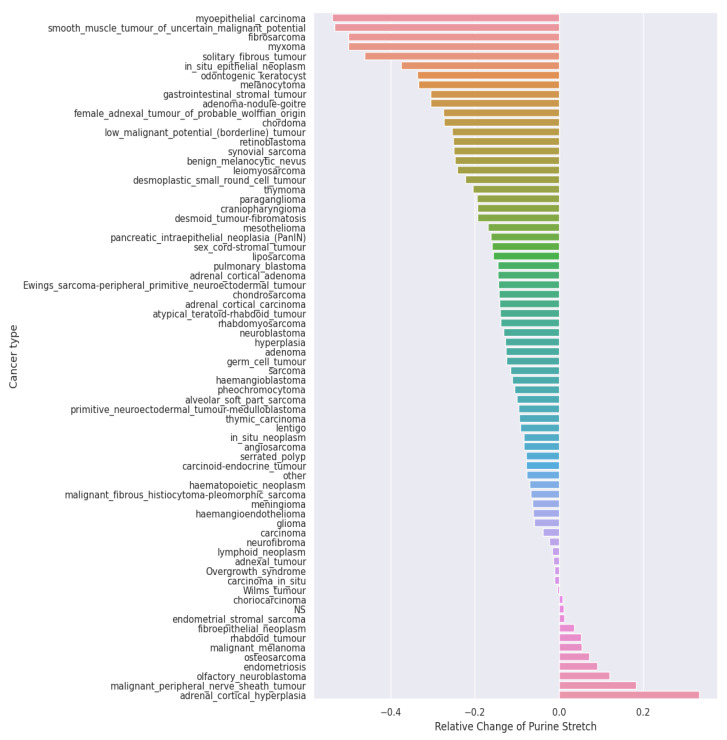
Distribution of the relative change in the purine chain in different types of cancer. The X axis is the relative change in the purine chain (Rel_Change_Pu), and the Y axis is the cancer type. Rel_Change_Pu was calculated using the following formula: (normal-cancer)/(max(normal, cancer)), where “normal” and “cancer” are corresponding Purine_chain_lengths. A positive value indicates a shortening of the purine chain in cancer compared to normal tissue, while a negative value indicates an extension.

We looked at the intergenic part of the promoters (400 bp upstream of the transcription start site). For the outcome metric, we used Rel_Change_Pu (relative change in Purine_stretch_length), which was calculated using the following formula: (normal-cancer)/(max(normal, cancer)), where “normal” and “cancer” are corresponding Purine_chain_lengths. Rel_Change_Pu was measured separately for each type of cancer (Figure 6).

In this analysis, Rel_Change_Pu varied greatly depending on the type of cancer. In most cancer types, Rel_Change_Pu was negative, which means that the purine chains in the cancer were elongated. However, there are also cancer types (e.g., melanoma) in which purine chains are more frequently shortened.

### 2.4. The Comparison of Purine_Stretch_Length with the Prevalence of Each Mutation in Cancer

Next, we explored two metrics: Purine_stretch_length, the primary measure of the purine stretch, and the prevalence of each mutation in cancer, defined by the number of patients within the dataset where the specific mutation occurred. For context, many mutations in the dataset were unique, occurring only once, while others appeared twice (i.e., in 2 out of 1233 patients) or in a greater number of patients. Mutations with a prevalence of 1 largely consist of random occurrences, likely resulting from cancer rather than causative factors. Mutations with prevalences of 2, 3, or 4 typically correspond to causative cancer factors but are not under robust positive selection by clonal cancer evolution. In contrast, mutations with a prevalence greater than 4 are typical cancer mutations, subject to strong selection.

In purine stretches, point substitution mutations can result in three types of alterations: breaks, joins, and purine synonymous substitutions. We implemented the BRETORA metric to quantify the frequency of breaks, with “BRETORA” standing for “BREaks TO all RAtio”. This metric is defined as the ratio of the number of breaks to the total count of all mutations.

**Figure 7:**
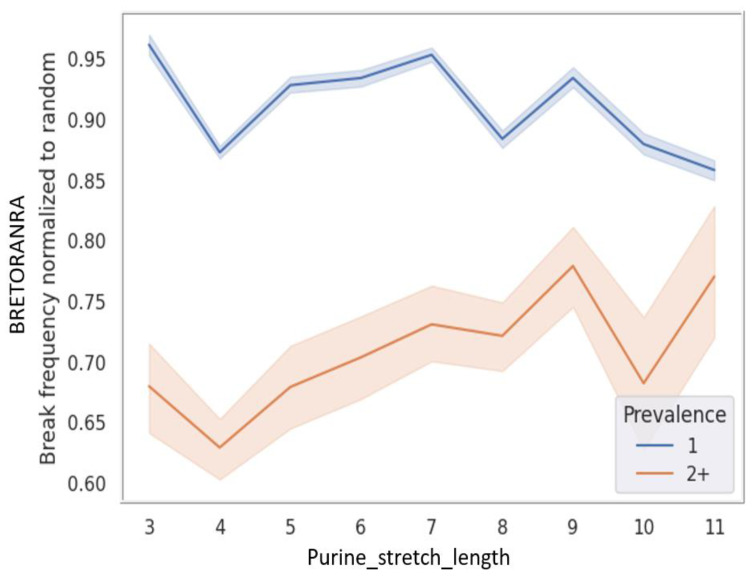
Typical cancer mutations avoid purine stretches. Break frequency normalized to random (BRETORANRA) versus mutation prevalence is plotted. The dotted blue line represents mutations with Prevalence 1; the dotted orange line represents mutations with Prevalence 2+. Shades represent the 95% percentile interval.

In examining purine chains, we aimed to measure the frequency of breaks in relation to chain length, specifically focusing on the functional influence we believe is associated with electron fusion in the aromatic rings of purines. However, longer purine stretches tend to be more frequently affected by random mutations, a factor we needed to exclude to obtain an accurate measurement. To normalize the data and exclude the random influence of length, we generated a random control by simulating 1,000,000 mutations. A summary table was then produced, presenting BRETORA values as functions of both prevalence and Purine_stretch_length. These values were normalized to the random ones, creating a new metric called BRETORANRA, short for “BRETORA Normalized to RAndom”. Figure 7 shows a graph depicting the dependence of this parameter on chain length for Prevalence 1 and 2+ mutations, reflecting our targeted analysis of chain length’s influence.

The findings presented in Figure 7 reveal that common cancer mutations with a prevalence of 2+ exhibit a notably lower normalized frequency of breaks when contrasted with primarily random mutations characterized by a prevalence of 1. This difference is particularly pronounced in shorter purine stretches.

## 3. Discussion

Purine stretches, composed of adenine (A) and guanine (G), serve crucial roles in DNA binding and gene regulation, and any mutations or alterations within these regions could potentially influence cancer development [1,2,3,4,5].

This is further supported by the aromatic nature of purines and their electron delocalization, factors that influence DNA structure, and their specific stacking energies could make these stretches more susceptible to specific mutations or alterations relevant to cancer.

Potential delocalization of electrons in purine stretches could also play a protective role against damage, and any disruption to this could make DNA more susceptible to damage, leading to mutations commonly found in cancer. Furthermore, the nature of the damage that causes mutations in purine stretches in cancer could be speculated upon. Cytosine deamination in promoters can be ruled out as a contributing factor, as it primarily causes transition mutations (C > T), which do not create or lengthen purine stretches. Other mechanisms, such as oxidative damage or replication errors, might be more relevant and should be investigated further. Understanding these specific mechanisms could provide deeper insights into the role of purine stretches in cancer development.

Additionally, the flanking sequences’ influence and the distance-decaying relationships extending up to 2000 nucleotides might impact how a mutation in or near a purine stretch affects adjacent regions, possibly relating to the proliferation or suppression of cancerous cells. Understanding the electron behavior in purine stretches using quantum chemical models could provide novel insights or therapeutic strategies, emphasizing the multifaceted nature of purine stretches in cancer.

## 4. Materials and Methods

### 4.1. Dataset of Mutations in Cancer

The data source used in this work was the CosmicNCV.tsv dataset, which was downloaded from the COSMIC website (https://cancer.sanger.ac.uk/cosmic/download, (accessed on 6 October 2024)). From it, we obtained the positions of mutations in the non-coding part of the genome in cancer patients and their diagnosis. For each mutation, information is given about the patient (identifier, organ where the cancer occurred, type of cancer, and cancer histology). The mutations are paired per patient. For each patient, a reference allele from the normal tissue and the mutant allele from the cancer tumor are available in the dataset. The dataset contains a total of 18 M mutations in 158 thousand patients (the average number of mutations per patient is 117). A subset of 4.7 M mutations in promoter regions was selected for analysis.

### 4.2. Total Lengths of Genes, Introns, Exons, and Repetitive DNA

The human genome version GRCh38 (release 108) was downloaded from the Ensembl site (https://ftp.ensembl.org/pub/release-108/fasta/homo_sapiens/dna/ (accessed on 6 October 2024)). The annotation of the human genome in gtf format was also downloaded from the Ensembl website (https://ftp.ensembl.org/pub/release-108/gtf/homo_sapiens/ (accessed on 6 October 2024)). Genomic regions were analyzed using the following algorithm. In the first step, the file with the annotation of the genome was parsed, and the coordinates of the beginning and end of all genes, all exons, and all UTRs for chromosomes 1–22 + X, Y were written out in separate dictionaries. The coordinates of introns were obtained by finding the gaps between exons, and the coordinates of intergenic sites were obtained by finding the gaps between genes. Since genes in the genome overlap, it was necessary to combine all overlapping coordinates and remove nested coordinates so that the same coordinate was not counted twice when calculating site lengths. This was done using the merge_regions() and drop_nested() functions from the Genome_Regions_Analysis.ipynb notebook. Then, after merging and clearing of nesting, the sum of coordinates within all the lists was calculated. The fraction N of nucleotides was calculated by finding the coordinates of all features in the genome and counting the fraction N within these sites. The type of exon in which N is present was also taken from the gtf annotation.

### 4.3. Obtaining the Type of Mutation and Length of the Purine Stretch

In the first stage of the work, information on the effect of the mutation on the purine chain, which includes the mutated nucleotide, was obtained. To do this, for each mutation, its position on the chromosome was found. Then, the chromosome segment flanking this mutation was taken with a shoulder length of 60 nucleotides (60 on the left, 60 on the right = total length of the segment of 120 nucleotide pairs). All sequences were converted to the purine code (purines A, G = R; pyrimidines T, C = Y). If the mutated nucleotide was a purine, the analysis continued on this DNA strand. If the mutated nucleotide was a pyrimidine, the analysis continued on the complementary DNA strand (R was replaced by Y and Y by R). If both the reference and mutant alleles were of the same type (purines or pyrimidines), such a mutation was labeled as purine synonymous. Otherwise, the type of mutation was determined as follows. If the mutation occurred within the purine chain (purines to the left and right of the mutated nucleotide—RRR), then the type of mutation was designated as a break. If there were pyrimidines to the left and right of the mutation, such a mutation restored the purine chain, and its type was designated as a join (YRY). If there was a pyrimidine on the left of the mutation and a purine on the right, such a mutation switched the purine chain, and its type was designated as LeftFlip (YRR). If there was a purine to the left of the mutation and a pyrimidine to the right, such a mutation was designated as RightFlip (RRY). After determining the type of mutation, the length of the purine chain, which includes the mutated nucleotide, was determined before and after the mutation (Ref_Chain_Length and Alt_Chain_Length, respectively). For this purpose, the length of the left and right arms (purine sequences to the left and right of the mutated nucleotide: left_shoulder and right_shoulder) was first calculated. Then, the lengths of the reference and mutant chains were calculated using the following formulas.

**Break type (RRR):**

Ref_Chain_Length = left_shoulder + right_shoulder − 1

Alt_Chain_Length = left_shoulder − 1 if left_shoulder ≥ right_shoulder else right_shoulder − 1

**Join type (YRY):**

Ref_Chain_Length = left_shoulder − 1 if left_shoulder ≥ right_shoulder else right_shoulder − 1

Alt_Chain_Length = left_shoulder + right_shoulder − 1

**LeftFlip type:**

Ref_Chain_Length = left_shoulder − 1 if left_shoulder − 1 ≥ right_shoulder else right_shoulder

Alt_Chain_Length = left_shoulder if left_shoulder + 1 ≥ right_shoulder else right_shoulder − 1

**RightFlip type:**

Ref_Chain_Length = left_shoulder if left_shoulder + 1 ≥ right_shoulder else right_shoulder − 1

Alt_Chain_Length = left_shoulder − 1 if left_shoulder ≥ right_shoulder else right_shoulder”

Then, for each mutation, the characteristic of the relative change in the purine chain (Rel_Change_Pu) was calculated using the following formula: (x − y)/(max(x,y)), where x is Ref_Chain_Length and y is Alt_Chain_Length. Then, all identified characteristics, including mutation type, Ref_Chain_Length, Alt_Chain_Length, and Rel_Change_Pu, as well as the primary purine chain sequence in which the mutation is included (Primary_Sequence) were recorded in the dataframe under analysis (CosmicNCV).

Also, it was additionally determined whether the mutation is included in the transcription start site (TSS). For this purpose, the origin coordinates of all genes were listed from the annotation file (Homo_sapiens.GRCh38.108.gtf). RNA-coding genes were not excluded, so the set of TSSs included TSSs from both protein- and RNA-coding genes. The fragment 400 nucleotides upstream the TSS was taken as the promoter. Then, for each mutation, it was determined whether it belongs to one of the promoters, and this information was also added to the dataframe being analyzed (as well as information about which gene that promoter belongs to, if the mutation belongs to it).

All the above procedures were done for each chromosome individually using the SNPU_w_syn.py script. The results for all chromosomes were then combined into one common dataset.

### 4.4. Relative Change of the Purine Stretch Length Analysis

The analysis of the obtained dataframe was performed in Jupyter Notebook (Project Jupyter, Python 3.14, Python Software Foundation, Wilmington, DE, USA), using pandas (Open source project), numpy (NumFy Project), scipy, matplotlib (Matplotlib Development Team), and seaborn (Open source project) libraries. The basic statistics of the dataframe (mean, standard deviation, median, and quartiles) were obtained using the pandas describe() method. The statistical significance of differences in Purine_stretch_length was calculated using the *t* test and Mann–Whitney from scipy.stats. To ensure the robustness of our findings, we employed several statistical approaches:

1. Normality Testing: Before applying *t* tests, we assessed the normality of our data using the Shapiro–Wilk test. For datasets that significantly deviated from normality, we employed non-parametric alternatives such as the Wilcoxon signed-rank test.
2. Paired Analysis: We utilized paired statistical tests to compare purine stretch lengths in tumor and normal tissue from the same patient, effectively controlling for patient-specific confounding factors.
3. Multiple Testing Correction: To account for multiple comparisons, we applied the Benjamini–Hochberg procedure to control the false discovery rate.
4. Effect Size Calculation: In addition to *p* values, we calculated Cohen’s d to quantify the magnitude of the observed differences.
5. Subgroup Analysis: We performed separate analyses for different cancer types and genomic regions to account for potential confounding effects of tumor heterogeneity.
6. Randomization Control: We created a randomized control dataset to compare against our observed results, helping to distinguish true biological effects from statistical artifacts.

### 4.5. Purine Stretch Break vs. Join Analysis (BRETORA)

The Break/Total ratio (BRETORA) as a function of Purine_stretch_length (Wire_Length) and mutation frequency (prevalence) was measured using the following algorithm. First, the number of times each mutation occurs in the dataset was counted (using value_counts() and the unique mutation identifier HGVSG). Then, the following analysis was performed for each prevalence from 1 to 8. From the dataframe containing mutations with a given prevalence, mutations in the purine chain of a certain length (3 to 17) were filtered out. Then, for these mutations, the number of breaks and the ratio of breaks to all mutation types (BRETORA) were counted. Thus, for each combination of prevalence and Wire_length values, the ratio of breaks to all other mutations (BRETORA) was obtained. Based on this, a summary table was made, and graphs (heatmap, lineplot) were plotted using the Seaborn library.

### 4.6. Randomized Control

In order to check the significance of the result, a random control was performed. To do this, the coordinates of all genes in the human genome were first written out from the annotation file. Then, 1,000,000 random numbers ranging from 1 to the chromosome length (mutation position) were simulated for each chromosome. It was then checked to see if this mutation fell into the position of one of the genes that were found in the previous step. If it does not, then it is an intergenic mutation, and further analysis is performed. Next, a random nucleotide was chosen where the substitution occurred. Then, all the simulated mutations were combined into one dataframe. In this dataframe, all the steps described in the previous paragraphs were performed, until a summary table containing the BRETORA values depending on prevalence and wire length was obtained.

### 4.7. Cancer Prevalence Analysis (PrevRa)

The BRETORA ratio of Prevalence 2+ mutations to BRETORA of Prevalence 1 mutations was analyzed. This characteristic was called PrevRa. This analysis was performed for the 10 most frequent cancer types in the dataset. For each cancer type, the analysis described in the BRETORA analysis item was first performed. Then, the PrevRa ratio was calculated from the summary table, and a heatmap was constructed reflecting this PrevRa value for each Purine_stretch_length in each cancer type.

## 5. Conclusions

Building upon these concepts, the investigation into the role of purine stretches in intergenic regions using the Cosmic dataset has revealed avoidance of purine stretches in intergenic regions by cancer mutations in many types of cancer. This avoidance was also dependent on the type of cancer.

The study also found a distinct shortening of purine stretches in cancerous tissue compared to normal tissue, reflecting potentially different strategies that cancer cells might use to modulate gene expression. This tendency for common cancer mutations to avoid purine stretches hints at the possible functional significance of purine stretches for cancer initiation, clonal expansion, or progression.

In this study, we found a statistical shortening of purine stretches in cancer tumors, providing evidence that purine stacks play a significant role in chromatin organization and gene regulation. The importance of aromatic stacking was a key motivation for our research, and our findings suggest that the stability provided by purine stacking influences the observed mutational patterns. The shortening of purine stretches likely disrupts these stabilizing interactions, leading to changes in chromatin structure. While our results do not directly measure aromaticity, the dependence on purine stretch length supports the hypothesis that purine stacking is crucial for maintaining DNA stability and function.

These findings collectively support the hypothesis that purine stretches in promoters have functional importance, especially in complex gene regulation functions and chromatin restructuring. The variations in purine stretches associated with different cancer types may contribute to unique disease pathways, highlighting the functional importance of intergenic sequences and their electronic structures.

Our computational analysis provides a foundation for understanding purine stretches in cancer. Future experimental studies, such as targeted mutagenesis, DNA melting experiments, or spectroscopic analyses, could directly measure purine stretch properties and their alterations in cancer. Such work would complement our computational approach and further elucidate the role of purine stretches in cancer development.

Overall, the results provide evidence for the importance of purine stretches in cellular function and suggest a complex relationship with different types of cancer. This research is crucial in unraveling the function of delocalized electron structures in intergenic regions and particularly in cancer.

## Data Availability

The original contributions presented in the study are included in the article; further inquiries can be directed to the corresponding authors. The authors partially presented data at The 3rd International Electronic Conference on Biomolecules organized by the MDPI journal *Biomolecules* that took place virtually on 23–25 April 2024.
